# Patients’ perceptions of interactions with hospital staff are associated with hospital readmissions: a national survey of 4535 hospitals

**DOI:** 10.1186/s12913-018-2848-9

**Published:** 2018-01-29

**Authors:** Lianping Yang, Chaojie Liu, Cunrui Huang, Dana B. Mukamel

**Affiliations:** 1 School of Public Health, Sun Yat-sen University, Guangzhou, China; 2 Global Health Institute, Sun Yat-sen University, Guangzhou, China; 3 School of Health Management, Hubei University of Chinese Medicine, Wuhan, China; 4 School of Psychology and Public Health, La Trobe University, Melbourne, Australia; 5 Department of Medicine, Division of General Internal Medicine, University of California, Irvine, Irvine, USA

**Keywords:** Hospital readmissions, Hospital staff, Patients’ perception, Responsiveness

## Abstract

**Background:**

Reducing 30-day hospital readmissions has become a focus of the current national payment policies. Medicare requires that hospitals collect and report patients’ experience with their care as a condition of payment. However, the extent to which patients’ experience with hospital care is related to hospital readmission is unknown.

**Methods:**

We established multivariate regression models in which 30-day risk-adjusted readmission rates were the dependent variables and patients’ perceptions of the responsiveness of the hospital staff and communication (as measured by the Hospital Consumer Assessment of Healthcare Providers and Systems (HCAHPS) scores) were the independent variables of interest. We selected six different clinical conditions for analyses, including acute myocardial infarction (AMI), chronic obstructive pulmonary disease (COPD), heart failure, hip/knee surgery, pneumonia, and stroke. Data included all acute care hospitals reporting in Hospital Compare in 2014.

**Results:**

The number of hospitals with reported readmissions ranged from 2234 hospitals for AMI to 3758 hospitals for pneumonia. The average 30-day readmission rates ranged from 5.19% for knee/hip surgery to 22.7% for COPD. Patient experience of hospital-staff responsiveness as “top-box” ranged from 64% to 67% across the six clinical conditions, communication with nurses ranged from 77% to 79% and communication with doctors ranged from 80% to 81% (higher numbers are better). Our finding suggests that hospitals with better staff responsiveness were significantly more likely to have lower 30-day readmissions for all conditions. The effect size depended on the baseline readmission rates, with the largest effect on hospitals in the upper 75th quartile. A ten-percentage-point increase in staff responsiveness led to a 0.03–0.18 percentage point decrease in readmission rates. We found that neither communication with physicians nor communication with nurses was significantly associated with hospital readmissions.

**Conclusions:**

Our findings suggest that elements of care related to staff responsiveness during patients’ stay may influence rehospitalization rates. Changes in staff responsiveness may offer an additional tool for hospitals to employ ongoing efforts to achieve reductions in readmissions, an important objective both financially and for patient health outcomes.

**Electronic supplementary material:**

The online version of this article (10.1186/s12913-018-2848-9) contains supplementary material, which is available to authorized users.

## Background

Reducing 30-day hospital readmissions has become an important focus of the current national payment policies in the U.S. [1–3]. Reduction of hospital readmission rates is essential to contain unnecessary health care costs and improve the quality of inpatient care. One in five Medicare beneficiaries is readmitted within 30 days, at a cost of more than $26 billion per year [1, 4]. Furthermore, readmissions lead to significant burdens not only on the healthcare system, but also on individual patients.

Many readmissions are considered avoidable if the care provided in the preceding admission was of high quality. The Readmission Reductions Program implemented by the Centers for Medicare and Medicaid services (CMS) in October 2012 penalizes hospitals financially if their readmission rates exceed pre-specified standards [5]. Risk adjusted readmissions also serve as an indicator of the quality of hospital care and are published in the Hospital Compare web-based report card [6].

With these powerful incentives hospitals have been adopting various approaches to lower readmissions. Hansen and colleagues reviewed and categorized these interventions into three domains: pre-discharge interventions, post-discharge interventions, and interventions that serve as a “bridge” across care settings and are implemented both before and after patient discharge [7]. Pre-discharge interventions include medication reconciliation, patient education, discharge planning, and formulating a follow-up appointment before discharge. Post-discharge interventions include coordination and management, such as follow-up phone calls to patients, timely communicating with ambulatory health service providers and timely home-based visits [7, 8]. Bridging interventions include physician continuity crossing the outpatient- and inpatient-settings, guided transition by coaches, [9] and hospital discharge instructions designed according to patient centered care [7]. The evidence about the effectiveness of these interventions is, however, mixed. While many interventions are effective at reducing readmissions, the more effective ones are complex and include supporting patients’ capacity for self-care [1]. In general, no single intervention or measure implemented alone was found to be associated with reduced risk for readmissions [7]. The fact that, although many factors outside of the hospital contribute to unplanned readmissions, many readmissions occur within 30 days of discharge, implies that there is room for improvement.

One of the areas that has not been investigated is the relationship between patients’ communications with the hospital staff, staff responsiveness to patients, and readmission rates. We hypothesized that hospitals where staff maintain better communication and responsiveness, not only at the time of discharge but throughout the stay would achieve lower readmission rates for two reasons: 1) the rapport achieved during the stay between the staff and the patient is likely to improve the “efficiency” and effectiveness of any education and communication attempted during the post-discharge or bridging processes discussed above; and 2) there may be a residual effect of educational and/or behavioral effect imparted during the inpatient stay that influences the patient behavior, including compliance with discharge instruction. This may not be exhibited by patients who did not experience a supportive and responsive relationship with their medical staff during their inpatient stay.

To test this hypothesis we present an analysis of the association between hospital readmission rates and patients’ perceptions of their relationship with the hospital staff in terms of responsiveness and communication. Our measures of responsiveness and communication were taken from the Hospital Consumer Assessment of Healthcare Providers and Systems (HCAHPS) survey [10]. The HCAHPS is a survey mandated by the Centers for Medicare & Medicaid Services (CMS) for the U.S. acute care community hospitals. It is administered to a sample of surgical, medical, and obstetric patients. The survey measures nine key domains of hospital care quality: communication with doctors, communication with nurses, hospital-staff responsiveness, management of pain, communication on medicines, information of patient discharge, hospital cleanliness and quietness, overall rating of the hospital, and patient’s willingness to recommend the hospital [11–13]. Patients’ experiences of hospital care have been shown to be associated with patient safety indicators and measures of technical process [13]. However, there is paucity in the literature documenting the relationship between patient’s perceptions of hospitalization care and hospital readmissions [7].

## Methods

### Data and sample

This study included the 4535 all acute care and critical access hospitals, Medicare certified U.S. hospitals that were reported on in the December 2014 Hospital Compare CMS web-based report card. They represent 79.8% of all hospitals in the U.S.

Hospital Compare is a web-based quality report card published by CMS [6]. It includes in addition to the HCAHPS data discussed above other hospital information including clinical quality measures such as risk-adjusted mortality rates and 30-day readmission rates, measures based on adherence to evidence-based guidelines, and other general information about the hospital such as ownership, hospital type and services provided.

### Variables

The dependent variable was 30-day readmission rate as reported in Hospital Compare. The 2014 Hospital Compare reports these rates for six clinical conditions: acute myocardial infarction (AMI), chronic obstructive pulmonary disease (COPD), heart failure (HF), hip/knee surgery (HK), pneumonia (PN), and stroke (STK)). All were reported for the patients admitted during the July 2010 through June 2013 period.

There were three independent variables of interest, all based on HCAHPS: staff responsiveness, doctor-patient communication, and nurse-patient communication. We chose these three indicators because they are more likely to capture the response of doctors/nurses to patients’ requests. Other indicators such as “communication about medicine”, “discharge information” or “care transition” were excluded in data analyses because they are more likely to be initiated by doctors/nurses rather than patients.

The HCAHPS is a risk-adjustment survey of patients’ experiences during the period of hospitalization. It is administered in several different ways (e.g. face-to-face, phone, mail, or combination), to a random sample of hospitalized adult patients between 48 h and 6 weeks after their discharge from the hospital. The HCAHPS includes 32 questions overall: 4 screening questions, 7 demographic items, and 21 questions about the patient’s experience while in the hospital. The HCAHPS data are adjusted by CMS for survey mode (mail only, telephone, mixed) and patient characteristics. The responses are calculated and shown as percentage of surveyed patients, and the feedback responses are categorized into three levels. For example: “nurses always; nurses usually; or nurses sometimes/never; communicated well.” The highest possible response of patients in each domain is called the “top box” response. The “top box” ratings for each hospital are presented in the online report card, which is reported publicly on the CMS Hospital Compare web site. The 2014 Hospital Compare contains HCAHPS data for inpatient discharges between January 2013 and December 2013.

We included three “top box” HCAHPS responses. Staff responsiveness is measured in HCAPHS on the basis of two questions: “(1) After you pressed the call button, how often did you get help as soon as you wanted it?” and “(2) How often did you get help in getting to the bathroom or in using a bedpan as soon as you wanted?” The two other variables capture (separately) doctor and nurse communication with patients through responses to three questions: “How often do doctors (or nurses) (1) treat you with courtesy and respect? (2) listen carefully to you? (3) explain things in a way you could understand?” The responses to the individual questions are combined by Hospital Compare into three composite measures: a staff responsiveness measure; a doctor-patient communication measure and a nurse-patient communication measure, which are then each reported as “top box” in Hospital Compare. In our analyses we tested the associations between the three “top box” responses and hospital readmissions.

We also included several control variables which may influence hospital readmission rates. We included dichotomous variables indicating hospital ownership (Government as the reference category, For-profit and Non-profit), hospital type (Acute Care Hospitals as the reference category, or Critical Access Hospitals), and provision of emergency services (or not as the reference category).

### Statistical analysis

We performed multivariate regression analyses in which the unit of analyses was the hospital. We estimated separate linear regression models for each of the six clinical conditions for readmissions, and included the staff responsiveness, communication with doctor and communications with nurse variables in each. Inference was based on robust standard errors with clustering at the state level. The initial analyses suggested that the relationship between patients’ perceptions of their relationship with staff and hospital readmissions may not be linear. We, therefore, stratified the hospitals into three groups based on readmission rates: the lowest quartile (25th quartile), the 25th–75th quartiles, and the highest quartile (75th quartile). The large sample size allowed us to split the sample so that we could use linear regression analyses to identify determinants of hospital readmissions and determine potential ceiling effect of responsiveness/communication on hospital readmissions. All analyses were repeated separately for each stratum. We used Stata version 13.1 for all statistical analyses.

Because our hypotheses, posited above, are all directional (e.g. better responsiveness lowers readmissions), we used one-sided tests to evaluate them. We rejected statistical significance if *p* > 0.05.

### Ethical approval

This study was based on hospital level, publicly available information. We did not collect, analyze, or report any individual level, identifiable, human subject’s data. Hence this study is not considered human subject research. It was approved by the Institutional Review Board of the School of Public Health, Sun Yat-sen University.

## Results

Table 1 describes the characteristics of the study hospitals. For each clinical condition, the sample is different because not all hospitals have their data reported for the condition due to small numbers. The number of hospitals with recorded readmissions ranged from 2234 hospitals for AMI to 3758 hospitals for pneumonia.

**Table 1:** Descriptive statistics: readmission rates, staff responsiveness, nurse or doctor communication, and hospital Characteristics by clinical condition

Variables	Acute myocardial infarction	Chronic obstructive pulmonary disease	Heart failure	Hip/knee surgery	Pneumonia	Stroke
	Mean(SD)	Mean(SD)	Mean(SD)	Mean(SD)	Mean(SD)	Mean(SD)
Sample size	2234	3513	3574	2734	3758	2740
30-day readmission rate (%)	17.84(1.06)	20.76(1.31)	22.70(1.66)	5.19(0.67)	17.35(1.31)	13.30(1.20)
Percent of patients reported in the Top Box for the category						
Responsiveness of hospital staff (%)	63.78(6.24)	66.43(7.91)	66.53(7.96)	65.98(7.54)	67.03(8.35)	64.77(6.71)
Communication with nurse (%)	77.32(4.42)	78.44(4.99)	78.47(5.02)	78.41(4.69)	78.70(5.24)	77.76(4.52)
Communication with doctor (%)	79.60(3.84)	81.19(4.88)	81.24(4.93)	80.60(4.14)	81.45(5.12)	80.29(4.29)
Hospital characteristics						
Hospital ownership	%	%	%	%	%	%
Government	12.85	18.96	19.11	14.63	20.65	15.58
For-profit	18.53	16.94	17.04	19.60	16.37	17.66
Non-profit	68.62	64.10	63.85	65.76	62.99	66.75
Hospital type	%	%	%	%	%	%
Acute Care Hospitals	98.93	84.26	83.52	92.28	80.39	94.31
Critical Access Hospitals	1.07	15.74	16.48	7.72	19.61	5.69
Hospital Offers Emergency Services(yes)	98.39	98.29	98.18	96.42	98.08	98.65

There was considerable variability in readmission rates and patient-reported experiences with hospital staff. The mean of 30-day risk-adjusted readmission rates ranged from 5.19 to 22.70% across all clinical areas, representing a more than fourfold variation. However, the variation across hospitals within each clinical condition was substantially lower, with the highest coefficient of variation (standard variation as a percentage of the mean) being 13% for hip/knee surgery. There was much less variation in staff responsiveness and communication across the six clinical conditions. Responsiveness ranged from 64 to 67% across the six clinical conditions, communication with nurses ranged from 77 to 79% and communication with doctors ranged from 80 to 81%. Variation across hospitals was similar to readmissions. The largest coefficient of variation for all was for pneumonia at 13% for responsiveness, at 7% for communication with nurses and at 6% for communication with physicians.

The 18 regression models are presented in the Additional file 1. We found that neither communication with physicians nor communication with nurses was significantly associated with hospital readmissions. However, staff responsiveness was, and these results are summarized in Table 2.

**Table 2:** Associations between patients’ perceived responsiveness of hospital staff and 30-day readmission for all 6 clinical conditions by hospital readmission rate

Variables	Hospital stratum by readmission rates					
	lowest quartile		25th–75th quartile		highest quartile	
	Coefficient	*P*-value	Coefficient	P-value	Coefficient	P-value
Acute myocardial infarction	−0.001	0.448	**−0.010**	**0.002**	**−0.014**	**0.013**
Chronic obstructive pulmonary disease	− 0.004	0.138	**− 0.009**	**0.001**	**− 0.018**	**0.006**
Heart failure	0.001	0.550	**−0.006**	**0.048**	−0.006	0.269
Hip/knee surgery	0.005	0.929	**−0.003**	**0.013**	**−0.011**	**0.016**
Pneumonia	0.003	0.753	**−0.007**	**0.002**	−0.007	0.146
Stroke	0.007	0.894	−0.006	0.080	**−0.018**	**0.048**

Bold datas indicate *p*<0.05

Note: Results are summarized from 18 regression models. Full results are shown in the appendix

*P* values are for the one tailed hypothesis that higher responsiveness is associated with lower 30-day readmission test

**Fig. 1:**
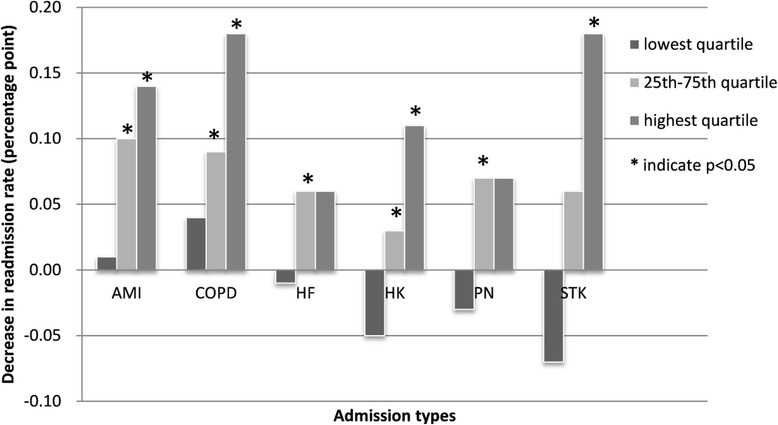
Decrease in readmission rate due to 10 percentage points increase in staff responsiveness. AMI: acute myocardial infarction, COPD: chronic obstructive pulmonary disease, HF: heart failure, HK: hip/knee surgery, PN: peneumonia, STK: stroke

Furthermore, Fig. 1 depicts the decrease in 30-day readmission rates due to a 10-percentage point’s increase in staff responsiveness. Patients’ perceived responsiveness of hospital staff is a significant predictor of lower unplanned 30-day risk-adjusted readmission rates for all six clinical conditions, except for hospitals with the lowest readmission rates, in the bottom quartile.

For AMI, COPD and hip/knee surgery, it is a significant predictor for both hospitals in the middle range and those with the worst readmission rates. For the other three conditions, the effect reached significance only for one of the two groups, but the trend was the same - i.e. better staff responsiveness was associated with lower readmissions. We also note that except for heart failure and pneumonia, the effect was much larger for those hospitals in the worst quartile of readmissions.

## Discussion

This study was designed to contribute to our understanding of the factors that are associated with hospital readmissions. The high percent of 30-day readmissions among Medicare patients, which hovers around 20%, presents both a financial and quality of care concern. It has recently become a focal point of interest to hospital physicians and management as they are held accountable both financially, through the Medicare payment system, and “reputationally” through the Hospital Compare reporting system.

The Hospital Readmissions Reduction Program, implemented in October 2012, is intended to change hospital behavior by enhancing financial incentives; exactly how hospitals should respond to these financial incentives is not quite clear. Our study was motivated by the extant literature, suggesting that a highly responsive staff provides higher quality care, averts adverse outcomes, and hence is likely to also prevent readmissions. Some studies have shown that there is an association between patient’s perceptions of hospital-staff responsiveness and quality of hospital care [13, 14]. Saman et al. disclosed a significant association between lower hospital-staff responsiveness and higher rates of a hospital acquired infection - central line associated bloodstream infections [14]. Another study of 4605 hospitals demonstrated that hospitals with higher rates of pressure ulcers are less likely to receive a “top box” rank for timely hospital-staff responsiveness [15]. The hospitals are also less likely to achieve a “top box” rank for timely hospital-staff responsiveness, if the hospitals have higher rates of postoperative death from deep venous thrombosis, and treatable complications [15]. Staff responsiveness may also prevent falls and other sequelae, by offering patients help out of bed as soon as requested. Such adverse events increase further readmission risk [16].

Indeed we found that hospitals with better staff responsiveness had lower 30-day readmission rates. We estimate that a 10-percentage point improvement in hospital-staff responsiveness is associated with a 0.03 to 0.18 percentage point decrease in readmission rates, depending on the condition and the hospital initial readmission level. The small effect size may be associated with the small variations of staff responsiveness (independent variable) and hospital readmissions (dependent variable) in our data. The variation of hospital readmissions within each clinical condition is very low, with the highest coefficient of variation being 13% for hip/knee surgery. Overall, patient perceived hospital-staff responsiveness is quite low, only around 65% (ranging from 64 to 67%). The low level of perceived hospital-staff responsiveness and low variations in both independent and dependent variables may have limited our ability to detect the actual effect size of staff responsiveness on hospital readmissions. We also tested interactions between the independent variables (for example between staff responsiveness and hospital type) in the regression modeling. But all of the models pointed to a potential ceiling effect within the current range of hospital staff-responsiveness. For hospitals that are already operating at very high levels of performance vis-a-vis readmissions – i.e. the bottom quartile of readmission rates – our findings suggest that there is no benefit from further increasing staff responsiveness. Only hospitals which are at the 25th–75th percentile and upper 75th percentile of readmissions have an advantage from increasing staff responsiveness. In fact, the hospitals with the worst readmission performance, at the highest quartile of rates, are the ones that are likely to gain the most, for all but two of the conditions we studied.

What might be the processes behind the associations between staff responsiveness, and low readmission rates that we observed? Prompt staff responsiveness to patient-initiated call buttons is a critical component of the patient experience during a hospital stay. The call light could be a lifeline for hospitalized patients. Hospital responsiveness depends largely on its nursing staff [17]. Poor staff responsiveness could be attributed to three main causes: process design, communication problems, and staffing issues. Process design involves a lack of policies and procedures for handling call buttons, perhaps an absence of teamwork-based approach by staff to answer call buttons [18]. Communication issues include delays in relaying patients’ requests to the primary registered nurse or nurse assistant, while the patient is unaware that staff is performing other tasks [18]. Staffing related issues are associated with staff shortages [19]. Findings from previous studies indicate that hospitals with better manageable and accountable nurse workloads (e.g. higher levels of registered nurse (RN) staffing in the hospital) face lower hospital readmissions rates [17, 20–22].

The cross-sectional design of this study requires caution in drawing conclusion from this study on how to improve practice. This study, does, however, suggest further areas for study, that would investigate any causal relationship between patients’ and staff responsiveness to communication with patients on the one hand and lower readmissions rates on the other hand. Some of the specific questions raised by this study include: (1) To what extent does fast staff response time lower re-admission rates? And why does it differ across conditions? (2) Does patient understanding and satisfaction play an equally important role in lowering re-admission rates? (3) What resources and measures will need to be mobilized to improve staff responsiveness to patients’ questions and communication needs? Does it require a culture change in hospitals, which in all likelihood is not easy to accomplish? (4) What differences are there in patient behaviors between those who have different perceptions while in the hospitals and after hospital discharge? (5) Is patient case mix and patient Socio-demographic important?

Hospital readmission rates change as a result of the Readmission Reductions Program by CMS. Such a change can be shaped by various factors, including those that occur before and after hospital discharge. It is important to note that patients’ experience is a direct indicator of the quality of hospital care. Patients’ experience in hospitals can also impose a profound impact on their post-discharge care. This is simply because discharged patients need to follow a discharge plan developed by their hospital doctors and nurses. Further studies should adopt a longitudinal design to determine the predictive value of patients’ experience on readmission rates. The Hospital Compare should also report additional variables.

If future studies support our findings and suggest changes in staff responsiveness may offer an additional tool for hospitals to employ as viable strategies in lowering rehospitalization rates, hospitals will have to assess how resource intensive such a transformation is and whether it is worth the benefit in terms of improved performance. As such, future studies should evaluate not only the benefits due to lower readmissions, but also the benefits due to improved quality during the stay itself as well as the “costs” that hospitals might encounter when trying to change staff behavior.

Hospitals may choose from a menu of approaches to improve the responsiveness of hospital staff. An obvious, albeit a costly strategy, is increasing staffing level. Such a strategy may, therefore, not be adopted by hospitals without further external incentives such as mandated minimum staffing levels adjusted for patient case-mix, or benchmarking and financial incentives according to nurse staffing, or public reporting of nursing workforce levels [20]. California in the U.S. is the only state that has set up stringent requirements on how many patients that nursing staff can take care for at a given unit interval [20]. Hospitals may also consider instituting changes in nurse work flows. Evidence has shown that hourly rounding programs improve patients’ perceptions of nursing responsiveness [18]. Hourly rounding improves nursing responsiveness, which in turn reduces patients’ risk for adverse events, such as falls and complications, and ultimately readmissions [16].

Unlike our hypothesis about staff responsiveness, our hypotheses about the association between better communication and lower readmission rates were not confirmed by the analyses for any of the conditions we studied, neither for communications with physicians nor for communications with nurses. This is surprising because the literature does suggest that communication between providers and patients is associated with better outcomes [15, 23–25].

There are several limitations in this study. In the HCAHPS, responsiveness and communication is measured by multiple questions. However, due to limited details in the Hospital Compare report, we were not able to perform further analyses on the correlation between each measured item and readmission rates. The available data did not allow us to perform subgroup (e.g. age and gender) analyses either. However, the patient perception indicators were adjusted for differences in age and gender.

## Conclusions

Elements of care related to staff responsiveness may contribute to quality of care and to lower readmission rates. Changes in staff responsiveness may offer an additional tool for hospitals to employ ongoing efforts to achieve reductions in readmissions, an important objective both financially and for patient health outcomes. Hospitals may choose from a menu of approaches to improve the responsiveness of hospital staff, such as increasing staffing level, and instituting changes in nurse work flows.

## Additional file

Additional file 1: Multivariable regressions - Predictors of 30-day readmission rates by clinical conditions. Details of the 18 multivariate regression models are presented in the supplementary materials. (DOCX 31 kb)

## Abbreviations

AMI: Acute myocardial infarction
CMS: The Centers for Medicare and Medicaid services
COPD: Chronic obstructive pulmonary disease
HCAHPS: The Hospital Consumer Assessment of Healthcare Providers and Systems
HF: Heart failure
HK: Hip/knee surgery
PN: Pneumonia
STK: Stroke
