# Supplementary material for: Patients’ perceptions of interactions with hospital staff are associated with hospital readmissions: a national survey of 4535 hospitals
Source: BMC Health Serv Res. 2018 Jan 29;18:50. doi: 10.1186/s12913-018-2848-9 (PMC5789545; doi:10.1186/s12913-018-2848-9)
Supplement: Supplementary file 1 — Multivariable regressions - Predictors of 30-day readmission rates by clinical conditions. Details of the 18 multivariate regression models are presented in the supplementary materials. (DOCX 31 kb) [file 12913_2018_2848_MOESM1_ESM.docx]

**Supplementary Tables**

**Supplement to: Patients’ perceptions of interactions with hospital staff are associated with hospital readmissions: A national survey of 4535 hospitals**

**Supplementary Tables: Multivariable regressions - Predictors of 30-day readmission rates by clinical conditions**

| **Table S1. Predictors of 30-day readmission for acute myocardial infarction by hospital readmission rate** | | | | | | |
| --- | --- | --- | --- | --- | --- | --- |
| Variables | Hospital stratum by readmission rates | | | | | |
|  | lowest quartile | | 25^th^-75^th^ quartile | | highest quartile | |
|  | Coefficient | P-value | Coefficient | P-value | Coefficient | P-value |
| Responsiveness of hospital staff* | -0.001 | 0.448 | **-0.010** | **0.002** | **-0.014** | **0.013** |
| Communication with nurses* | 0.007 | 0.692 | 0.009 | 0.960 | 0.009 | 0.771 |
| Communication with doctors* | 0.002 | 0.589 | -0.003 | 0.271 | -0.004 | 0.327 |
| Hospital ownership (ref: Government) |  |  |  |  |  |  |
| For-profit | -0.088 | 0.363 | -0.012 | 0.752 | -0.001 | 0.991 |
| Non-profit | -0.062 | 0.403 | -0.026 | 0.343 | -0.041 | 0.661 |
| Hospital type (ref: Acute care hospitals) |  |  |  |  |  |  |
| Critical Access Hospitals | 0.224 | 0.024 | -0.056 | 0.253 | -0.411 | 0.000 |
| Emergency service provided | 0.017 | 0.951 | 0.122 | 0.121 | 0.181 | 0.017 |
| Constant | 15.874 | 0.000 | 17.697 | 0.000 | 19.360 | 0.000 |

* P <0.05 was considered statistically significant using one-tailed test.

| **Table S2. Multivariable predictors of 30-day readmission for chronic obstructive pulmonary disease** | | | | | | |
| --- | --- | --- | --- | --- | --- | --- |
| Variables | Hospital stratum by readmission rates | | | | | |
|  | lowest quartile | | 25^th^-75^th^ quartile | | highest quartile | |
|  | Coefficient | P-value | Coefficient | P-value | Coefficient | P-value |
| Responsiveness of hospital staff* | -0.004 | 0.138 | **-0.009** | **0.001** | **-0.018** | **0.006** |
| Communication with nurses* | 0.010 | 0.916 | 0.009 | 0.980 | 0.004 | 0.629 |
| Communication with doctors* | 0.008 | 0.917 | 0.001 | 0.623 | 0.010 | 0.726 |
| Hospital ownership (ref: Government) |  |  |  |  |  |  |
| For-profit | -0.033 | 0.655 | 0.027 | 0.418 | 0.003 | 0.979 |
| Non-profit | -0.072 | 0.159 | 0.020 | 0.446 | 0.094 | 0.333 |
| Hospital type (ref: Acute care hospitals) |  |  |  |  |  |  |
| Critical Access Hospitals | 0.203 | 0.000 | -0.031 | 0.353 | -0.242 | 0.003 |
| Emergency service provided | -0.219 | 0.099 | -0.017 | 0.785 | 0.160 | 0.384 |
| Constant | 18.498 | 0.000 | 20.436 | 0.000 | 22.077 | 0.000 |

* P <0.05 was considered statistically significant using one-tailed test.

| **Table S3. Multivariable predictors of 30-day readmission for heart failure** | | | | | | |
| --- | --- | --- | --- | --- | --- | --- |
| Variables | Hospital stratum by readmission rates | | | | | |
|  | lowest quartile | | 25^th^-75^th^ quartile | | highest quartile | |
|  | Coefficient | P-value | Coefficient | P-value | Coefficient | P-value |
| Responsiveness of hospital staff* | 0.001 | 0.550 | **-0.006** | **0.048** | -0.006 | 0.269 |
| Communication with nurses* | 0.001 | 0.522 | 0.007 | 0.846 | -0.013 | 0.181 |
| Communication with doctors* | 0.014 | 0.925 | -0.003 | 0.263 | 0.010 | 0.804 |
| Hospital ownership (ref: Government) |  |  |  |  |  |  |
| For-profit | -0.052 | 0.576 | -0.012 | 0.840 | 0.234 | 0.062 |
| Non-profit | -0.141 | 0.107 | -0.071 | 0.031 | 0.166 | 0.039 |
| Hospital type (ref: Acute care hospitals) |  |  |  |  |  |  |
| Critical Access Hospitals | 0.433 | 0.000 | -0.066 | 0.077 | -0.455 | 0.002 |
| Emergency service provided | -0.207 | 0.214 | -0.139 | 0.276 | 0.286 | 0.151 |
| Constant | 19.818 | 0.000 | 22.972 | 0.000 | 24.638 | 0.000 |

* P <0.05 was considered statistically significant using one-tailed test.

| **Table S4. Multivariable predictors of 30-day readmission for hip/knee surgery** | | | | | | |
| --- | --- | --- | --- | --- | --- | --- |
| Variables | Hospital stratum by readmission rates | | | | | |
|  | lowest quartile | | 25^th^-75^th^ quartile | | highest quartile | |
|  | Coefficient | P-value | Coefficient | P-value | Coefficient | P-value |
| Responsiveness of hospital staff* | 0.005 | 0.929 | **-0.003** | **0.013** | **-0.011** | **0.016** |
| Communication with nurses* | -0.002 | 0.372 | 0.005 | 0.960 | 0.017 | 0.949 |
| Communication with doctors* | -0.002 | 0.285 | -0.002 | 0.177 | -0.004 | 0.301 |
| Hospital ownership (ref: Government) |  |  |  |  |  |  |
| For-profit | 0.031 | 0.407 | 0.017 | 0.463 | -0.039 | 0.589 |
| Non-profit | -0.021 | 0.544 | 0.005 | 0.727 | -0.064 | 0.252 |
| Hospital type (ref: Acute care hospitals) |  |  |  |  |  |  |
| Critical Access Hospitals | 0.190 | 0.000 | -0.029 | 0.222 | -0.197 | 0.000 |
| Emergency service provided | 0.229 | 0.027 | -0.003 | 0.941 | 0.017 | 0.876 |
| Constant | 3.899 | 0.000 | 5.136 | 0.000 | 5.790 | 0.000 |

* P <0.05 was considered statistically significant using one-tailed test.

| **Table S5. Multivariable predictors of 30-day readmission for pneumonia** | | | | | | |
| --- | --- | --- | --- | --- | --- | --- |
| Variables | Hospital stratum by readmission rates | | | | | |
|  | lowest quartile | | 25^th^-75^th^ quartile | | highest quartile | |
|  | Coefficient | P-value | Coefficient | P-value | Coefficient | P-value |
| Responsiveness of hospital staff* | 0.003 | 0.753 | **-0.007** | **0.002** | -0.007 | 0.146 |
| Communication with nurses* | -0.003 | 0.356 | 0.006 | 0.942 | 0.000 | 0.509 |
| Communication with doctors* | 0.006 | 0.869 | -0.002 | 0.232 | 0.001 | 0.546 |
| Hospital ownership (ref: Government) |  |  |  |  |  |  |
| For-profit | 0.083 | 0.263 | 0.003 | 0.923 | 0.006 | 0.951 |
| Non-profit | 0.012 | 0.810 | -0.012 | 0.667 | 0.009 | 0.869 |
| Hospital type (ref: Acute care hospitals) |  |  |  |  |  |  |
| Critical Access Hospitals | 0.120 | 0.013 | -0.011 | 0.736 | -0.364 | 0.000 |
| Emergency service provided | -0.169 | 0.066 | 0.076 | 0.143 | 0.542 | 0.000 |
| Constant | 15.599 | 0.000 | 17.245 | 0.000 | 18.382 | 0.000 |

* P <0.05 was considered statistically significant using one-tailed test.

| **Table S6. Multivariable predictors of 30-day readmission for stroke** | | | | | | |
| --- | --- | --- | --- | --- | --- | --- |
| Variables | Hospital stratum by readmission rates | | | | | |
|  | lowest quartile | | 25^th^-75^th^ quartile | | highest quartile | |
|  | Coefficient | P-value | Coefficient | P-value | Coefficient | P-value |
| Responsiveness of hospital staff* | 0.007 | 0.894 | -0.006 | 0.080 | **-0.018** | **0.048** |
| Communication with nurses* | -0.003 | 0.399 | 0.002 | 0.646 | 0.000 | 0.509 |
| Communication with doctors* | 0.008 | 0.884 | 0.001 | 0.565 | -0.005 | 0.337 |
| Hospital ownership (ref: Government) |  |  |  |  |  |  |
| For-profit | 0.211 | 0.002 | -0.022 | 0.482 | -0.058 | 0.502 |
| Non-profit | -0.032 | 0.561 | -0.080 | 0.030 | 0.043 | 0.672 |
| Hospital type (ref: Acute care hospitals) |  |  |  |  |  |  |
| Critical Access Hospitals | 0.200 | 0.001 | -0.072 | 0.027 | -0.257 | 0.048 |
| Emergency service provided | -0.091 | 0.467 | 0.061 | 0.568 | 0.322 | 0.060 |
| Constant | 11.133 | 0.000 | 13.267 | 0.000 | 15.64 | 0.000 |

* P <0.05 was considered statistically significant using one-tailed test.
